# Anusol Suppositories in Hemorrhoids, Etc.

**Published:** 1909-02

**Authors:** 


### ANUSOL SUPPOSITORIES IN HEMORRHOIDS, ETC.

Professor H. Strauss of Berlin, in a paper on "Proctitis Sphincterica" (*Hufeland' sche Gesellschaft,* May 14, 1908; *Berl. klin. Wochenschrift,* August 3, 1908) recommends a cellulose-free, non-constipating diet to insure feces devoid of coarse and mechanically irritating material. Friction of the feces at the sphincter should also be prevented by injection of oil or introduction of suppositories prior to defecation. In local therapy by means of astringent suppositories he accords the first place to the anusol suppositories. In severe cases, however, special topical treatment (silver nitrate, ichthyol, with the addition of eucain, etc.) is often also necessary.

